# Preparation and Characterization of Carboxymethyl Hydroxypropyl Cellulose

**DOI:** 10.3390/molecules31020387

**Published:** 2026-01-22

**Authors:** Meng He, Yanmei Lin, Yujia Huang, Xiuxing Ma, Yuanqiang Guo, Yuliang Ke, Huazhen Lai, Zhaopeng Wang, Zhanhua Chen, Xiaofang Zhang, Hangyu Dai, Mengna Feng, Yunhui Fang, Xiaopeng Xiong

**Affiliations:** 1 Lets Group, Xiamen 361001, China or hemeng666@ycit.edu.cn (M.H.); linyanmei@lets.com (Y.L.); maxiuxing@lets.com (X.M.); guoyuanqiang@lets.com (Y.G.); keyuliang@lets.com (Y.K.); laihuazhen@lets.com (H.L.); wangzhaopeng@lets.com (Z.W.); chenzhanhua@lets.com (Z.C.); zhangxiaofang@lets.com (X.Z.); 2 Key Laboratory for Advanced Technology in Environmental Protection of Jiangsu Province, School of Materials Science and Engineering, Yancheng Institute of Technology, Yancheng 224051, China; huang13913906481@163.com (Y.H.); 17768162533@163.com (H.D.); fmn0705@ycit.edu.cn (M.F.); 3 Department of Materials Science and Engineering, College of Materials, Xiamen University, Xiamen 361005, China; 4 Sichuan Province Engineering Technology Research Center of Novel CN Polymeric Materials, Chengdu 611731, China

**Keywords:** carboxymethyl hydroxypropyl cellulose, hydroxypropylation, salt tolerance

## Abstract

Carboxymethyl hydroxypropyl cellulose (CMHPC) combines the advantages of both carboxymethyl and hydroxypropyl substitutions, exhibiting superior solubility, viscosity characteristics, and enhanced salt tolerance compared to carboxymethyl cellulose (CMC). This study presents an optimized synthesis route for CMHPC through homogeneous hydroxypropylation of CMC under alkaline conditions. The effects of key reaction parameters, including propylene oxide amount and reaction time, on the structure and resulting properties were systematically investigated. The resulting CMHPC were comprehensively characterized using FTIR, solid state ^13^C NMR, and scanning electron microscopy (SEM), etc., confirming the successful hydroxypropyl group incorporation and morphological changes. In our findings, the suitable concentrations for NaOH and CMC were 5% and 4%, respectively, which could balance the yield and solution fluidity. CMHPC exhibited a much faster dissolution speed (3–5 min) than that of CMC (>30 min), indicating markedly enhanced hydrophilicity and solubility. Moreover, CMHPC also exhibited improved salt and acidity tolerance due to the steric hindrance of hydroxypropyl groups. CMHPC was also used to modify recycled coarse aggregate (RCA), and the results indicated that CMHPC could enhance the surface compactness and structural integrity of RCA. Moreover, CMHPC effectively improved the water resistance of RCA by constructing a physical barrier and optimizing the pore structure of the aggregate. This research provides valuable insights into the fabrication of modified cellulose ethers in homogeneous systems and offers a practical pathway for producing high-value cellulose derivatives with tailored properties, particularly for potential construction applications.

## 1. Introduction

Cellulose ethers (CEs) represent a class of semisynthetic polymers derived from natural cellulose, one of the most abundant renewable resources on Earth [1,2]. The structural backbone of CEs consists of β(1→4) linked D-glucose units, where the hydroxyl groups at positions 2, 3, and 6 undergo substitution with various functional groups. The resultant materials exhibit enhanced solubility and tailored physicochemical properties while maintaining the biodegradability and biocompatibility of cellulose [3,4]. The degree of substitution (DS) and molar substitution (MS) critically determine the solubility, viscosity, and the overall performance of the final products [5,6]. CEs are generally categorized into ionic types and non-ionic types, with each category exhibiting distinct behaviors in different environments. The global interest in CEs has grown substantially due to their versatile applications across numerous industries, including pharmaceuticals, food, cosmetics, petroleum, textiles, and construction [7,8,9].

Carboxymethyl cellulose (CMC), particularly in its sodium salt form (CMC-Na), stands as one of the most significant and widely produced ionic CE [6,10,11]. The production of CMC involves a two-step process consisting of the formation of alkali cellulose and carboxymethylation using monochloroacetic acid or its sodium salt. Its aqueous solution exhibits polyelectrolyte behavior, contributing to its functionality as a thickening and protective colloid. CMC has been widely used in the petroleum and paper industries, as well as in food products. Additional applications include the utilization of sizing agents in textiles, a thickener in printing pastes, and thickening agents and tablet binders in pharmaceuticals and cosmetics [6,7,10]. Despite its extensive utility, CMC exhibits certain limitations, particularly its sensitivity to multivalent cation environments and low pH conditions, where CMC solutions tend to form precipitates [12]. These constraints have motivated researchers to develop modified CEs with improved performance characteristics. This has led to the development of dual-functionalized derivatives such as carboxymethyl hydroxypropyl cellulose (CMHPC), which is produced through hydroxypropylation [13,14]. Hydroxypropylation introduces hydroxypropyl groups onto the cellulose backbone through a reaction with propylene oxide under alkaline conditions [15,16]. Compared to ionic cellulose ethers, such as CMC, the hydroxypropyl substitution offers distinct advantages. It can impart enhanced solubility in both aqueous and organic media, improved thermal gelation behavior, and reduced sensitivity to ionic strength and pH variations [17,18]. The improvement of solubility can be attributed to the steric hindrance effect from the hydroxypropyl groups, which disrupts the regular packing of cellulose chains. The incorporation of hydroxypropyl groups could possibly solve CMC’s weak tolerance to low pH and salt solutions, which is beneficial for applications in the building field.

The production and application of recycled coarse aggregate (RCA) from construction and demolition waste (CDW) offers an effective solution to CDW accumulation. This, in turn, substantially reduces reliance on the extraction of natural aggregate (NA) [19,20,21,22,23,24,25]. However, compared with NA, RCA exhibits inherent defects, including irregular particle shape with excessive angularity, rough surface texture, high water absorption, elevated porosity, and poor crushing resistance. These defects severely restrict the widespread application of RCA in high-performance construction scenarios [26,27]. To address these limitations, surface modification of RCA prior to its incorporation into concrete has become a critical research focus. Extensive studies have demonstrated that physical or chemical modification techniques can effectively mitigate the drawbacks and enhance the comprehensive performances of RCA [28,29]. It is noted that chemical modification typically involves treating RCA with admixtures (e.g., silane coupling agents, calcium silicate hydrate precursors, or polymer emulsions). These admixtures could form a dense coating on the RCA surface or fill internal pores, thereby reducing water absorption and enhancing the interfacial transition zone (ITZ) between RAs and the cement matrix [20,30,31,32]. The above modification strategies have been proven to significantly improve the properties of RCA to broaden its application in the construction industry. CEs can also form dense coatings due to their strong intermolecular interaction, and the utilization of CEs to modify the RCA is attracting [7].

Thus, a worthwhile endeavor would be the fabrication of CMHPC by using CMC as the raw material. In the present work, we tried to graft the hydroxypropyl groups onto the CMC chains in a homogeneous solvent system. We systematically studied the effects of the reaction time and the amount of propylene oxide on the structure and properties of CMHPC. The resultant CMHPC was used to modify RCA, hoping to broaden the potential application of CEs in the construction field.

## 2. Results and Discussion

### 2.1. Appearance and Morphologies of CMHPC

**Figure 1:**
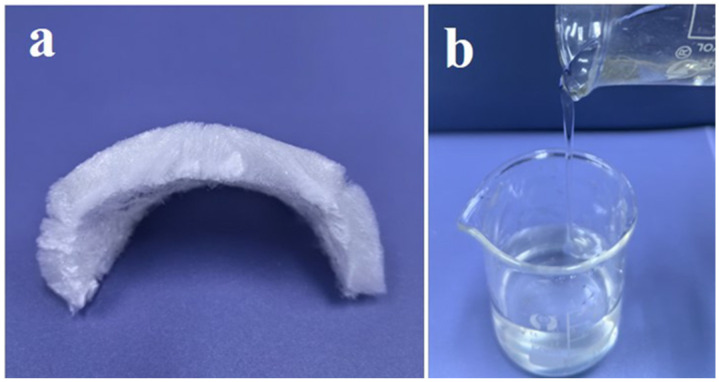
Photographs of the CMHPC-2 sponge obtained after freeze-drying (**a**) and the CMHPC-2 solution after dissolution (**b**).

The resulting CMHPC-2 solution was subsequently subjected to precipitation, drying, dialysis, and freeze-drying, successfully yielding a CMHPC-2 sponge (Figure 1a). This CMHPC sponge demonstrated rapid dissolution in distilled water within 3–5 min. The dissolution rate was significantly faster than that of the original CMC (>30 min), indicating markedly enhanced hydrophilicity and solubility. The dissolved CMHPC solution was clear and transparent (Figure 1b), with a relatively high viscosity.

**Figure 2:**
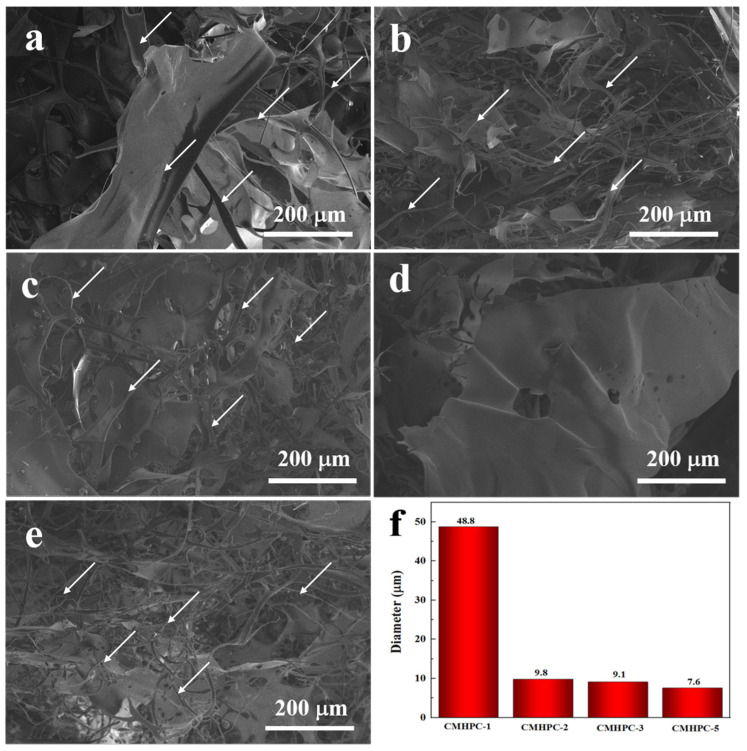
SEM images of CMHPC-1 (**a**), CMHPC-2 (**b**), CMHPC-3 (**c**), CMHPC-4 (**d**), CMHPC-5 (**e**), and the average fibril diameters (**f**). The white arrows indicate microfibrils.

A more hydrophilic polymer network likely leads to distinct ice crystal growth dynamics during freezing, ultimately forming a porous structure with smaller pores upon sublimation [33]. The effects of the reaction parameters, namely the amount of propylene oxide (the etherifying agent) and the reaction time, on the morphologies of the lyophilized CMHPC sponges were systematically studied using SEM. Figure 2 shows the microstructure of CMHPC sponges from different reaction conditions. Clearly, the microstructures of the sponges obtained under different reaction conditions varied, mainly in the sizes of the microfibrils. The average diameter of CMHPC fibrils decreased markedly from 48.8 μm to 9.8 μm with prolonged reaction time (1 h→2 h) (Figure 2a,b). The further reaction time (2 h→3 h) had only a little effect on the average diameter of fibrils (9.8 μm→9.1 μm). The size difference was possibly attributed to the different hydrophilicities of CMHPC, which directly affected the freezing behavior of the aqueous CMHPC solutions during the sponge preparation process. Furthermore, the sponge’s microstructure changed from sheet-like (Figure 2d) to a microfibrillar structure (Figure 2b,e) with increasing the etherifying agent molar ratios from 1.12 to 2.24. The average diameter of fibrils decreased from 9.8 μm to 7.6 μm (Figure 2b,e) with further increase in the molar ratio of etherifying agent to 4.46. Thus, both the amount of etherifying agent and the reaction time influenced the morphologies of the sponges.

### 2.2. Structure and Properties of CMHPC

**Figure 3:**
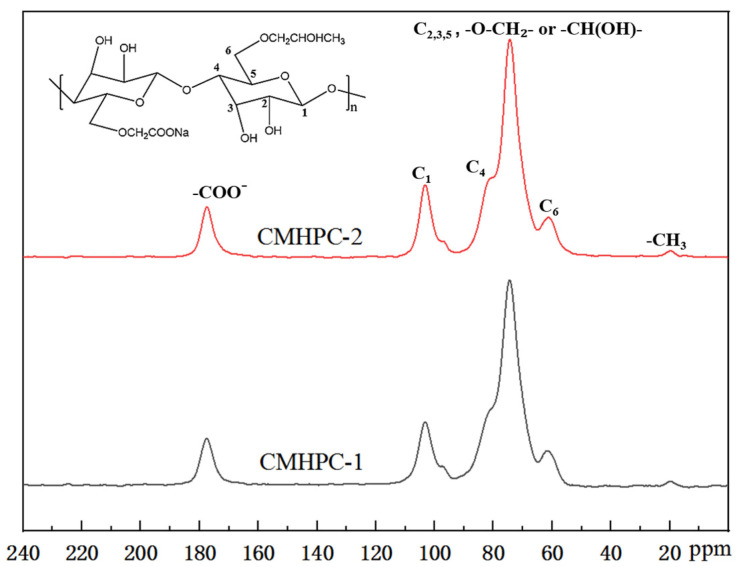
^13^C-NMR spectra of CMHPC-1 and CMHPC-2.

The hydroxypropylation of CMC was also evaluated by solid-state ^13^CNMR. Figure 3 shows the ^13^C-NMR spectra of CMHPC-1 and CMHPC-2. The signal at 177.5 ppm was assigned to the carbonyl carbon of the carboxymethyl group (-COO^−^), a signature of the CMC [6]. The resonances corresponding to the cellulose skeleton were observed at 103.1 ppm (C_1_), 81.2 ppm (C_4_), and a cluster of signals between 61.2 and 74.5 ppm, which were attributed to the C_2_, C_3_, C_5_, and C_6_ positions. The hydroxypropyl methylene group (-O-CH_2_-CH(OH)-CH_3_) is known to resonate in the 65–75 ppm region [34]. The signal observed at 74.5 ppm was therefore consistent with this methylene carbon, as well as with backbone carbons (C_2_, C_3_, C_5_), possibly substituted. Crucially, the appearance of a new resonance at 19.9 ppm, a characteristic of the terminal methyl group (-CH_3_) in the hydroxypropyl substituent, confirmed the successful hydroxypropylation.

**Figure 4:**
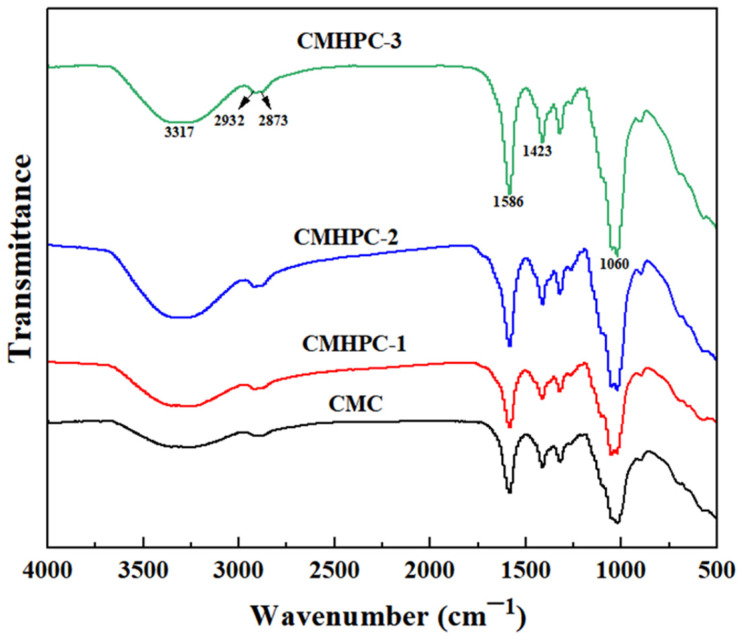
FTIR spectra of CMC, CMHPC-1, CMHPC-2, and CMHPC-3.

Figure 4 presents the FTIR spectra of CMC and CMHPC. Obviously, all samples exhibited absorption peaks at ~1420 cm^−1^ and 1590 cm^−1^, which were attributed to the symmetric stretching and asymmetric stretching of -COO^−^ groups [6], respectively. This indicated that the carboxymethyl substituents remained unaffected in CMHPC. Characteristic absorption peaks appeared at ~1060 cm^−1^, 2930 cm^−1^, and 3320 cm^−1^ in CMHPC, which were attributed to the stretching vibrations of -C-O-C-, C-H, and -OH, respectively. These results indicated the successful introduction of hydroxypropyl groups, confirming that hydroxypropylation occurred in CMC. Compared to CMHPC-1, the intensity of the peak at ~1060 cm^−1^ increased in CMHPC-2 and CMHPC-3, suggesting that a longer reaction time resulted in more hydroxypropylation.

**Figure 5:**
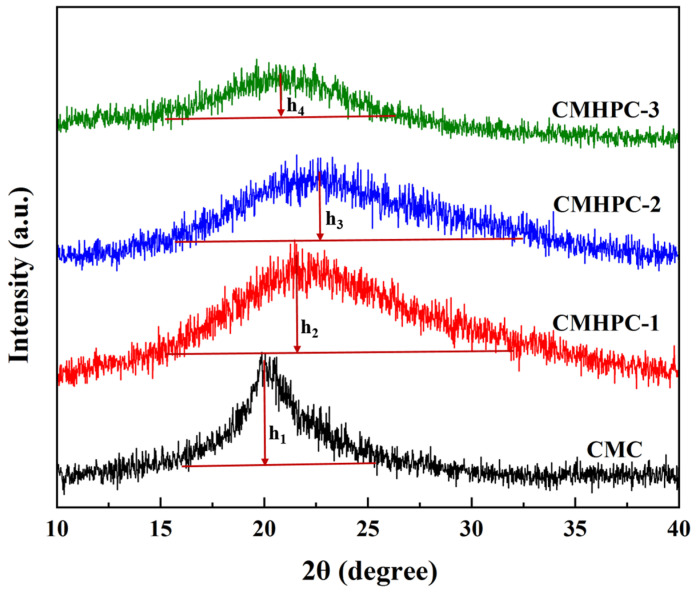
XRD patterns of CMC, CMHPC-1, CMHPC-2, and CMHPC-3.

XRD is commonly employed to characterize the crystalline structure of materials. For CEs, the change in characteristic peak intensity often reflects the extent of chemical modification because the introduction of ether groups typically disrupts the native crystalline order of cellulose. Figure 5 shows the XRD patterns of CMC, CMHPC-1, CMHPC-2, and CMHPC-3. There were no obvious peaks of cellulose in CMHPC-1, CMHPC-2, and CMHPC-3. Notably, the peak height of CMHPC-1 (h_1_) was 142% and 225% of that for CMHPC-2 (h_2_) and CMHPC-3 (h_3_), respectively. Meanwhile, the peak intensity of CMHPC-1 decreased gradually with the extension of reaction time. Moreover, the characteristic peak of CMC at 2θ = 20.2° shifted to ~22.0° for for CMHPC-1 and CMHPC-2. The weakening and amorphization of the diffraction features in CMHPC (on the basis of qualitative comparison of diffraction patterns rather than quantitative crystallinity analysis) indicated that the hydroxypropylation reaction effectively disrupted the short-range ordered packing of cellulose chains within the residual crystalline domains (rather than long-range crystallinity). The introduction of hydroxypropyl groups created steric hindrance and prevented the cellulose chains from packing into a well-defined lattice, thereby promoting a more amorphous structure and resulting in a change in characteristic peak position.

We have studied the solubility of CMC and CMHPC in different solutions, and the results are listed in Table 1. Sodium CMC is soluble in water due to the electrostatic repulsion of charged groups (-COO^−^) along the polymer backbone and promotion of extensive hydration via ion-dipole interactions with water molecules. CMC is soluble in NaCl solution because the interaction between Na^+^ from the NaCl and the CMC polyanion is non-specific and reversible. However, CMC was insoluble or poorly soluble in CaCl_2_, ZnCl_2_, and MgCl_2_ solutions due to the complex interplay of pH effects and specific cation-carboxylate interactions, which disrupt the polymer’s hydration and solubility [35]. As we expected, CMHPC-2 was soluble in all salt solutions due to the steric hindrance of hydroxypropyl groups, which physically obstructed the close coordination of carboxylate groups around different metal ions (including Na^+^, Ca^2+^, Zn^2+^, and Mg^2+^) [12]. Moreover, CMC is poorly soluble in HCl solution, while CMHPC-2 is soluble in HCl solution, which was also possibly due to the effect of the steric hindrance of hydroxypropyl groups.

**Figure 6:**
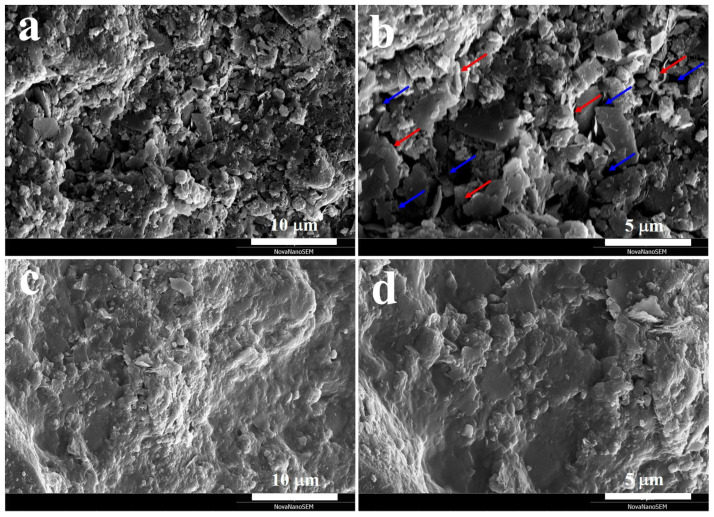
SEM images of RCA (**a**,**b**) and RCA-M (**c**,**d**). The blue and red arrows depict the pores and microcracks, respectively.

Figure 6 presents the SEM images of RCA and CMHPC-modified RCA (RCA-M). The surface of RCA (Figure 6a) was covered with numerous flaky and blocky residual mortar fragments as well as powdered cement paste. As shown in Figure 6b, noticeable pores and interconnected microcracks were observed on the surface, showing a loose and porous structure. This surface tends to form a wide and weakened ITZ between the RCA and the surrounding cement paste. Notably, the originally loose fine particles and open pores or cracks were largely covered and filled by the CMHPC modification layer (Figure 6c,d). Overall, the modification treatment effectively reduced the surface defect concentration and roughness of the RCA. CMHPC improved the surface compactness and structural integrity of RCA, which was conducive to reducing the porosity and the extent of weakening of the aggregate-cement ITZ.

**Figure 7:**
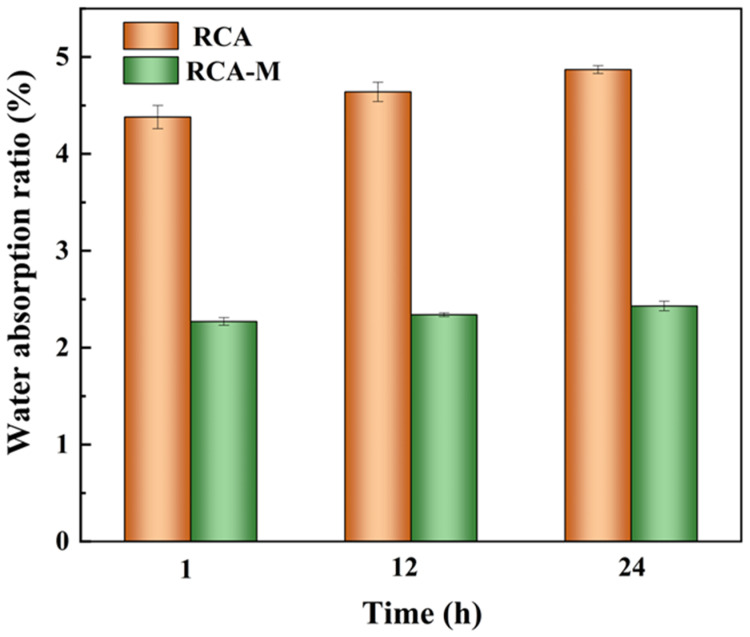
Water absorption ratios of RCA and RCA-M after 1 h, 12 h, and 24 h soaking.

The effect of CMHPC-2 on the RCA water absorption ratio was also evaluated. Figure 7 shows the water absorption ratios of RCA and RCA-M after 1 h, 12 h, and 24 h soaking. The water absorption ratios for RCA were 4.38%, 4.64%, and 4.87% after 1 h, 12 h, and 24 h soaking, respectively, which were relatively high due to their loose micro-structure (Figure 6). Notably, the water absorption ratios decreased significantly to 2.27%, 2.34%, and 2.43% for RCA-M at the same soaking time points, respectively. Thus, CMHPC modification effectively reduced the water absorption capacity of RCA. This phenomenon could be attributed to the intrinsic properties of CMHPC and its interaction with RCA. Specifically, CMHPC could form a continuous and dense hydrophobic film on the surface of RCA during the modification process (Figure 6). CMHPC not only covered the pores and microcracks on the aggregate surface but also penetrated partial internal pores. Consequently, the capillary channels, through which water permeates into the aggregates, were blocked. The water absorption ratio of RCA-M increased slightly with the extension of soaking time but remained at a low level overall. Overall, CMHPC modification improved the water resistance of RCA by constructing a physical barrier and optimizing the pore structure of the aggregate. It is noted that MS/DS have not been quantitatively determined and all structure–property correlations are based on relative trends inferred from reaction conditions and spectroscopic evidence.

## 3. Materials and Methods

### 3.1. Materials

CMC (degree of carboxymethyl substitution ~0.7), NaOH, glacial acetic acid, and acetone were obtained from Sinopharm Chemical Reagent Co., Ltd. (Shanghai, China). Propylene oxide was purchased from Aladdin Reagent Co., Ltd, Shanghai, China. All the above chemical reagents were of analytical grade and used without further purification. RCA was obtained from Huizhou Xinzhitong Construction and Development Co., Ltd, Huizhou, China.

### 3.2. Methods

#### 3.2.1. Preparation of CMHPC

In this experiment, CMC was used as the raw material to prepare CMHPC through an etherification reaction. We prepared different NaOH and CMC aqueous solutions and evaluated their fluidity to ensure the smooth progress of the etherification reaction. We found that the suitable concentrations for NaOH and CMC were 5% and 4%, respectively, which could balance the yield and solution fluidity. CMHPC sponge was obtained after post-treatment, with the specific operation process as follows: First, NaOH solution was prepared by completely dissolving NaOH in distilled water to obtain a 5 wt% NaOH aqueous solution. Next, desired amounts of CMC were weighed and slowly added to the above-prepared NaOH solution to form a transparent CMC-NaOH mixed solution with 4 wt% CMC content. Subsequently, the CMC-NaOH mixed solution was transferred to a reaction flask, which was heated to a constant temperature of 60 °C. A desired amount of propylene oxide was then slowly added dropwise in accordance with the experimental design, based on the molar ratios of propylene oxide/anhydroglucose unit (AGU) in CMC. The etherification reaction was conducted with continuous stirring at 60 °C for the desired reaction times. The “reaction time” was meticulously controlled from the moment the complete dose of propylene oxide was introduced into the carboxymethyl cellulose solution until the reaction was quenched. After the completion of the etherification reactions, the reaction products were naturally cooled to room temperature. Then, the reaction products were neutralized with acetic acid solution. The neutralized solution was slowly poured into a sufficient amount of acetone, while stirring during the pouring process, and white solid products gradually precipitated from the solution. The white products were filtered out and collected after the complete precipitation, which was then placed in an oven and dried at 60~80 °C until constant weight. Finally, the dried solid products were cut into small pieces and put into a pre-treated dialysis bag for complete dialysis purification to obtain purified CMHPC solutions. The purified CMHPC solutions were frozen and freeze-dried to obtain loose and porous CMHPC sponges. CMHPC sponges were coded as CMHPC-1, CMHPC-2, CMHPC-3, CMHPC-4, and CMHPC-5 according to the conditions listed in Table 2.

#### 3.2.2. Treatments of RCA Using CMHPC

RCA was thoroughly washed in clean water to remove soil impurities and loosely attached old mortar from the surface, then spread on metal trays and air-dried to constant mass. Then, the pretreated RCA was immersed in a prepared CMHPC-2 aqueous solution (CMHPC-2:water = 1:1000) for approximately 3 h, allowing the modifier to fully penetrate the surface pores of the aggregate. After soaking, the RCA was removed and drained for subsequent use, coded as modified RCA.

#### 3.2.3. Characterization of CMHPC

The surface morphology of the as-prepared CMHPC sponge was observed using a field emission scanning electron microscope (FE-SEM; Nova NanoSEM 450, FEI, Tokyo, Japan). For SEM analysis, the CMHPC sponge sample was directly sputtered with a thin gold layer to enhance electrical conductivity, and its morphology was observed directly via SEM. The average CMHPC microfibril diameters were calculated by measuring 20 microfibrils of each sample (from multiple SEM images) using Nano Measurer 1.2. The morphologies of unmodified RCA and modified RCA were also observed by SEM, which were coated with a layer of gold before observation. Fourier transform infrared (FTIR) spectra of the CMHPC sponge were recorded on an FTIR spectrometer (1600, Perkin-Elmer Co., Hopkinton, MA, USA) in the wavelength range of 4000–400 cm^−1^. The FTIR data were collected with a spectral resolution of 4 cm^−1^, accumulating 32 scans per spectrum. The CMHPC sponges for FTIR measurement were prepared using the KBr pellet method: a small amount of dried CMHPC sponge was ground into fine powder, which was then uniformly mixed with dried KBr at a certain mass ratio. The mixture was pressed into a transparent pellet using a tablet press under a specific pressure, and the pellet was used for the FTIR test.

Solid-state ^13^C nuclear magnetic resonance (^13^C NMR) spectroscopy was performed on two samples, designated CMHPC-1 and CMHPC-2. Prior to analysis, the samples were ground into a fine powder and oven-dried under vacuum for 48 h to remove residual moisture. Spectra were acquired on a Bruker AVANCE NEO 400 MHz spectrometer operating at a ^13^C resonance frequency of 75 MHz, using the cross-polarization magic-angle spinning (CP/MAS) technique. A MAS rate of 5 kHz was employed for both samples. The key acquisition parameters were as follows: a cross-polarization contact time of 3 ms, an acquisition time of 50 ms, and a relaxation delay of 3 s. Each spectrum was the result of 1024 accumulated scans to ensure an adequate signal-to-noise ratio. X-ray diffraction (XRD) patterns of CMC, CMHPC-1, CMHPC-2, and CMHPC-3 were recorded using an X’Pert3 Powder diffractometer (PANalytical, Almelo, The Netherlands) with Cu Kα radiation. Prior to measurement, both samples were ground into powders, placed in sample holders, and dried in a vacuum oven for 48 h to minimize the influence of moisture. Data were collected in the 2θ range of 5° to 40° at a scanning rate of 2°/min under ambient conditions. Phase identification was performed by comparing the diffraction patterns of CMHPC with the characteristic peaks of cellulose I and cellulose II [36,37]. The peak height and intensity, together with the peak position, were used to evaluate the hydroxypropylation of CMC.

CMC and CMHPC (0.25 g) were added to 50 mL of deionized water (25 °C) in a beaker placed on a magnetic stirrer, and the dissolution time was measured until the solutions became transparent without any discrete or settling particles. The solubility of CMC and CMHPC-2 sponges was also evaluated using different salt solutions, including NaCl saturated solution, CaCl_2_ (3.0 mol/L), ZnCl_2_ (3.0 mol/L), and MgCl_2_ (3.0 mol/L) solutions. Moreover, the solubility of CMC and CMHPC-2 sponges in 0.1 mol/L HCl solution was also evaluated. The solubility was denoted as “soluble” when the solution became transparent without obvious precipitate after 1 h of stirring and 15 min of standing. Meanwhile, the solubility was coded as “insoluble or poorly soluble” when most particles remained undissolved state or formed a precipitate on the bottom of a beaker under the same conditions.

Approximately 1000 g of RCA and RCA-M were weighed separately, which were then immersed in water-filled containers (the water level was kept about 5 mm above the surfaces of aggregates). The aggregates were taken out after soaking for 1 h, 12 h, and 24 h, respectively. The surface water was wiped off thoroughly before measuring their weights at the corresponding time points, which were denoted as W_1_. Subsequently, the aggregates were oven-dried to a constant weight and weighed again, with the obtained values recorded as W_2_. The water absorption ratios of RCA and RCA-M were calculated according to the following equation [38].

$$\mathrm{Water}\;\mathrm{absorption}\;\mathrm{ratio}\;\left(\%\right)=\frac{W_{1}-W_{2}}{W_{2}}\times 100\%$$

## 4. Conclusions

CMHPC was successfully homogeneously synthesized in an alkaline solution using CMC as a raw material. The suitable concentrations for NaOH and CMC were 5% and 4%, respectively, which could balance the yield and solution fluidity. CMHPC exhibited much faster dissolution speed (3–5 min) than that of CMC (>30 min), indicating markedly enhanced hydrophilicity and solubility. The amounts of etherifying agent and the reaction time influenced the morphologies of CMHPC sponges. Furthermore, CMHPC exhibited significantly improved tolerance to different salt solutions and acidic solutions, suggesting promising applications as concrete admixtures and in other industrial fields. CMHPC enhanced the surface compactness and structural integrity of RCA. Moreover, CMHPC modification improved the water resistance of RCA by constructing a physical barrier and optimizing the pore structure of the aggregate. This research provides valuable insights into the structure-property relationships of modified CE (CMHPC) and offers a potential practical pathway for producing high-value cellulose derivatives with tailored properties, particularly for construction applications such as RCA modification in concrete. It is noted that the current evaluation of RCA modification is mainly limited to microstructural observations and the water absorption ratio test; the mechanical performance of concrete incorporating modified RCA will be verified in our future work.

## Figures and Tables

**Table 1:** The solubility of CMC and CMHPC-2 in different salt and HCl solutions.

	Solutions	NaCl	CaCl_2_	ZnCl_2_	MgCl_2_	HCl
Samples						
CMC		+	-	-	-	-
CMHPC-2		+	+	+	+	+

+ Soluble, - insoluble or poorly soluble.

**Table 2:** Different conditions for the fabrication of CMHPC-1, CMHPC-2, CMHPC-3, CMHPC-4, and CMHPC-5.

Samples	Reaction Time (h)	Molar Ratios of Propylene Oxide/AGU
CMHPC-1	1	2.23
CMHPC-2	2	2.23
CMHPC-3	3	2.23
CMHPC-4	2	1.12
CMHPC-5	2	4.46

## Data Availability

The data that support the findings of this study are available from the corresponding author upon reasonable request.
